# Modern Approaches in the Discovery and Development of Plant-Based Natural Products and Their Analogues as Potential Therapeutic Agents

**DOI:** 10.3390/molecules27020349

**Published:** 2022-01-06

**Authors:** Asim Najmi, Sadique A. Javed, Mohammed Al Bratty, Hassan A. Alhazmi

**Affiliations:** 1 Department of Pharmaceutical Chemistry, College of Pharmacy, Jazan University, Jazan 45142, Saudi Arabia; anajmi@jazanu.edu.sa (A.N.); malbratty@jazanu.edu.sa (M.A.B.); haalhazmi@jazanu.edu.sa (H.A.A.); 2 Substance Abuse and Toxicology Research Centre, Jazan University, Jazan 45142, Saudi Arabia

**Keywords:** bioactivity-guided, drug discovery, extraction, isolation, plant-based natural products, ethnopharmacological

## Abstract

Natural products represents an important source of new lead compounds in drug discovery research. Several drugs currently used as therapeutic agents have been developed from natural sources; plant sources are specifically important. In the past few decades, pharmaceutical companies demonstrated insignificant attention towards natural product drug discovery, mainly due to its intrinsic complexity. Recently, technological advancements greatly helped to address the challenges and resulted in the revived scientific interest in drug discovery from natural sources. This review provides a comprehensive overview of various approaches used in the selection, authentication, extraction/isolation, biological screening, and analogue development through the application of modern drug-development principles of plant-based natural products. Main focus is given to the bioactivity-guided fractionation approach along with associated challenges and major advancements. A brief outline of historical development in natural product drug discovery and a snapshot of the prominent natural drugs developed in the last few decades are also presented. The researcher’s opinions indicated that an integrated interdisciplinary approach utilizing technological advances is necessary for the successful development of natural products. These involve the application of efficient selection method, well-designed extraction/isolation procedure, advanced structure elucidation techniques, and bioassays with a high-throughput capacity to establish druggability and patentability of phyto-compounds. A number of modern approaches including molecular modeling, virtual screening, natural product library, and database mining are being used for improving natural product drug discovery research. Renewed scientific interest and recent research trends in natural product drug discovery clearly indicated that natural products will play important role in the future development of new therapeutic drugs and it is also anticipated that efficient application of new approaches will further improve the drug discovery campaign.

## 1. Introduction

There is a long history of the usage of plant materials for treating human diseases. Several plant species such as opium (*Papaver somniferum*), myrrh (*Commiphora* species), and licorice (*Glycyrrhiza glabra*) have been mentioned on the clay tablets from Mesopotamia in 2600 BC; these plants are still used either alone or as one of the ingredients of herbal formulations for the treatment of various diseases. Furthermore, organic compounds from natural sources were used in the past and also used still to treat various diseases; these compounds are used in their natural form (as pure drug or phytomedicine) as well as serve as lead molecules for the development of synthetic and semisynthetic analogues with improved druggability. A number of such active constituents being in clinical application include morphine, codeine, noscapine, papaverine, quinine, artemisinin, paclitaxel, etc. [1,2,3].

In addition to the discovery of new chemical entities (NCEs) for therapeutic application, the natural products provide an important foundation as potential lead compounds for the development of new and more effective drugs through structural modification. Although natural products possess diverse and complex chemical structures, the plant secondary metabolites are seemed to exhibit greater biological friendliness and drug-likeness than those derived from purely synthetic sources. Consequently, the molecules from natural origin are supposed to be better candidates for further drug development [4,5]. Newman et al. (2003) reported that about 28% of all the new chemical entities (NCEs) entered into the market between 1981 to 2002 were from natural sources or natural product derived, and additionally, 24% of the NCEs were developed from chromophore analysis of the natural products [2]. The importance of natural products in modern medicine was further established by a report which mentioned that 23 natural products with diverse chemical structures found a place in the market between 2000–2005; in addition to many other NCEs under clinical trial phases of drug development [6].

The natural products and related drugs exhibit a broad spectrum of pharmacological activities, and they are used for the treatment and/or prevention of most of the popular human diseases, including infectious diseases, cancers, peptic ulcers, as immunomodulators, anticoagulants, antioxidants, respiratory, digestive and cardiovascular system-related diseases, antidiabetics, etc. [2]. After a thorough analysis of the prescription pattern in the United States of America, Grifo et al. (1997) reported that 84 of 150 prescription medicines were belonging to natural products and related drugs [7]. Patridge et al. (2016) analyzed the drugs approved by the United States Food and Drug Administration (USFDA) and found over one-third of all new molecular entities were natural products or their derivatives [8]. Out of 59 drugs approved by USFDA in 2018, approximately 16% were natural products or drugs inspired by natural products [9]. According to another report, in the years 2000, 2001, and 2002, of the highest 35 worldwide ethical drug sales, 40%, 24%, and 26%, respectively, accounted for natural products and drugs derived from natural products [10]. Paclitaxel, a plant-based anticancer drug is one of them [11].

About 35% of the annual global market of medicine is either from natural products or related drugs; mainly including plants (25%) followed by microorganisms (13%) and animal (3%) sources [12]. Between 1983 and 1994, the USFDA approved 520 new drugs, of that about 39% were natural products or drugs derived from them, while this proportion was about 60–80% in the case of antibiotics and anticancer agents [13,14,15]. Newman and Cragg (2016) reported that, of the 1562 drugs approved by USFDA between 1981 and 2014, 64 were pure natural products, 141 were herbal mixtures, 320 were derived from natural products, and 61 were synthetic drugs prepared by exploring the pharmacophores of natural products; these constitute 4%, 9.1%, 21%, and 4%, respectively, of the total approved drugs [16]. The examples of worldwide best-selling natural products derived medicines include antibiotics and antifungal agents, erythromycin, clarithromycin, amoxycillin, amphotericin B; anticancer agents, paclitaxel, docetaxel, and campothecin; cholesterol-lowering drugs, atorvastatin, simvastatin, lovastatin; immunosuppressant, tacrolimus, and cyclosporin A and antihypertensive agents, captopril, and enalapril [14,15,17].

There is a great requirement to stimulate the enthusiasm of the scientific research fraternity towards the incorporation of natural products in the drug development process. However, the major concern related to natural products is the success rate during the various drug development stages, mainly in the case of a random selection of the sources. In order to increase the success rate, the use of a well-designed strategy is necessary for the selection and shortlisting of the candidate species for investigation. A well-documented knowledge of phytomedicines, as in the traditional system of medicine or ethnopharmacological knowledge of herbal medicines, may be helpful to overcome the problems of low success rate and decrease the cost, and time of natural product development, which are considered to be the main associated factors [18,19]. The historical and traditional experiences of therapeutic application of plant materials have greatly assisted the isolation of a single chemical entity and its introduction into the modern system of medicine. Ethnopharmacological uses of the plant constituted as one of the most important sources of lead compounds for the early stages of drug discovery and development. Indeed, Fabricant and Farnsworth (2001) reported that about 80% of the 122 plant-derived natural products were related to ethnopharmacological uses [20]. Some important natural compounds derived from plant and microbial sources, along with their chemical structure and therapeutic indications, have been represented in Table 1.

The new chemical entities (NCEs) can be discovered from one of the four major natural sources including plants, marine, animals, and microorganisms (fungi and bacteria). In addition to these natural sources, the new compounds can also be prepared by using synthetic chemistry and combinatorial chemistry. Among the natural sources mentioned above, plants (botanical) source is of considerable importance and the present article is describing the approaches in the context of this source, with the main emphasis given to the bioactivity-guided fractionation approach. The natural compounds obtained from botanical sources may belong to one of the classes including, biologically active compounds directly used as therapeutic agents, lead compounds (with specific biological activity) for development of more potent analogues, compounds whose structures may provide novel pharmacophore that can be converted to druggable compounds or chemical entities to be used as a marker for standardization of crude plant extracts. Additionally, plant extracts can be used to develop herbal formulations (Herbal products).

**Figure 1:**
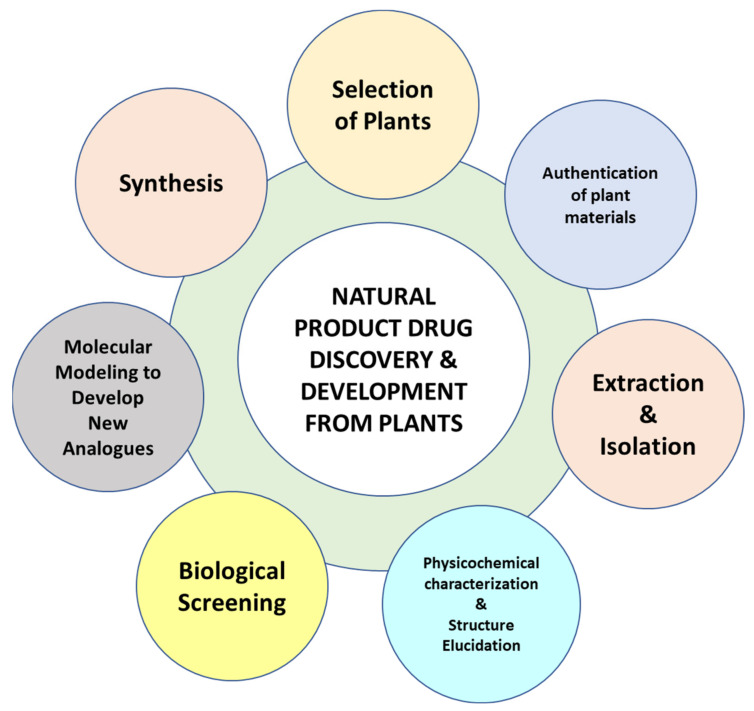
The major processes involved in the discovery and development of natural products from botanical sources.

Generally, the compounds isolated from natural sources possess some unique structural characteristics including, a greater number of oxygen atoms, more chiral centers, higher steric complexity, and molecular rigidity, a greater number of hydrogen bond acceptors and donors, and low aromatic ring atoms to total heavy atoms ratio. Moreover, there is a broader range of molecular properties including partition coefficient, molecular mass, and diversity of ring systems [3]. Owing to these unique characteristics of the compounds from natural sources, the development of analogues either to improve potency and pharmacokinetic properties or reduce toxicity is never an easy task for medicinal chemists. This article will highlight the modern approaches used for the discovery of natural products from botanical origin by following the proper methods of candidate selection, bioactivity-guided extraction, and fractionation, biological screening, phytochemical characterization to identify potential lead compounds for specific biological activity, and finally development of analogues of the identified lead through in-silico study and virtual screening techniques (Figure 1).

## 2. Selection of Plants for Screening

In the process of drug discovery from plant sources, the first and one of the most important steps involves the selection of plant candidates for extraction/isolation of active principles and screening for biological activities. According to Fabricant and Farnsworth (2001), out of approximately 250,000 available species of higher plants, only 15% were subjected to phytochemical screening and about 6% of the plant species were evaluated for their biological properties [20]. One or more of the following approaches described by them are generally being followed by researchers worldwide for this purpose.

### 2.1. Selection Based on Ethnopharmacological Knowledge

The discovery of new biologically active compounds using this approach of plant selection depends on the empirical experiences related to the use of the plants. The approach is based on the ethnomedicinal usage history of the plants, for example, andrographolide was isolated from the plant *Andrographis paniculta*, which was used for the treatment of dysentery in ethnomedicine. Moreover, a number of active constituents, including berberine, morphine, and picroside from *Berberis aristate*, *Papaver somniferum*, and *Picrorrhiza kurroa* were isolated through this approach. In this approach, the candidate plants are being selected on the basis of observation, description, and even some experimental evaluation. It may involve the study of botany, chemistry, pharmacology, biochemistry, archaeology, anthropology, and the historical background of the plant [18].

### 2.2. Random Approach

In this approach, mainly the plants are selected randomly from the local/national regions, and screening of the selected plants for target bioassays is performed. In addition to that, any of the target chemical classes of compounds such as flavonoids, alkaloids, polysaccharides, etc., may also be screened. This approach is used for focused as well as general screenings and provides a good chance of success. It is simple to select the plant candidates through this approach, however, disadvantageous in the sense that it does not provide any prior information regarding the biological activity of the selected species.

### 2.3. Approach Based on Traditional System of Medicine

Countries such as China and India have a rich heritage of well-documented records of traditional/herbal medicines, which is based on a codified system of medicines from botanical sources. The codified system approach is newer than the ethnomedicinal practice. It differs from ethnomedicinal practices in the following three accounts; firstly, these codified systems made up the empirical practices on the strong conceptual fundamentals of pharmacology and human physiology, while the latter relies mainly on the empirical experiences. Secondly, the concept of pharmaceutical formulations was more developed in the traditional codified system as compared to ethnomedicinal practices, where the products were used mainly as crude extracts such as decoction and juices. The term standardization was common in the traditional system of medicine. Lastly, the ethnomedicinal practices are generally controlled by a small fraction of the community and are localized in nature; on the other hand, the traditional system is much institutionalized.

Some of the important examples of natural products discovered by adopting the approach based on the codified system of medicine include bacosides from *Bacopa monnieri*, artemisinin from *Artemesia alba*, boswellic acid from *Boswellia serrata*, and reserpine from *Rauwolfia serpentine* used as a memory enhancer, antimalarial, anti-inflammatory, and antihypertensive agents, respectively [18]. Comparative characteristics of different approaches for the selection of plant candidates have been illustrated in Table 2.

## 3. Authentication of Plant

Authentication of the collected raw materials is the basic starting point in the development of natural products. The authentication of plant materials may be achieved through the application of one or more of the methods involving taxonomic, macroscopic, microscopic, chromatographic, spectroscopic, chemometric, immunoassays, and DNA fingerprinting analysis. Depending on the type of adulterants and closeness of the chemical constituents, a simple method such as examining the organoleptic properties may be enough to authenticate certain drugs, while a highly sophisticated method may be required by some other drugs. Therefore, it is up to researchers to select a suitable procedure for the material of interest.

The first step of authentication of medicinal plants involves identification of the botanical origin and the scientific binomial name is to be determined [50].

Macroscopic identification is performed by comparing the organoleptic properties, including color, odor, taste, size, shape, fracture characteristics, surface properties, and texture of the plant material with standard reference material [50].

Microscopic method is generally used to differentiate and identify very similar medicinal plants. The method is fast, convenient, and involves the determination of internal structural features at tissue and cellular levels using a microscope. For this purpose usually ordinary light microscope is enough, however, a polarized and fluorescence microscope may also be used to increase the accuracy of the detection [51,52,53].

Chromatographic techniques including thin-layer chromatography (TLC), high-performance thin-layer chromatography (HPTLC), high-performance liquid chromatography (HPLC), and capillary electrophoresis (CE) are extremely useful for qualitative and quantitative analysis of natural products. Herbal medicines possessing volatile principles are analyzed by gas chromatographic technique (GC) [54,55]. TLC provides preliminary fingerprinting of the natural product, and it is advantageous due to simplicity and multiple sample analysis in a single run. Volatile constituents of the natural medicine provide required fingerprints that can be applied for the identification of the plants. CE is advantageous due to its good separation efficiency, the requirement of a relatively small amount of sample, and fast analysis [55].

As the genetic make-up of each plant species is unique and remains unaffected by environmental factors, age, and other conditions; *DNA barcoding* may offer reliable information for authentication and quality control of medicinal plants. DNA barcoding offers species-level identification of plants using a short DNA region (DNA barcode). Consequently, this technique is widely applied in research and industry for molecular identification and solves a variety of problems related to taxonomy and population genetics, prevention of illegal wildlife collection and trade, and quality assurance of food and medicinal products. In the field of medicinal plants, initially, DNA barcoding was used as a tool for identification only, however, in recent years, it has made remarkable progress and is widely applied for quality assurance with respect to authentication of a number of herbal products [56,57,58]. Recently, the first general DNA-based method of identification was introduced by British Pharmacopoeia (BP). It used *Ocimum tenuiflorum* L. (Lamiaceae) as an example and the method emphasized plant sampling, DNA extraction, barcode region, purification, amplification, and sequence reference database [59,60].

## 4. Extraction and Isolation of Natural Compounds Using Biological-Activity Guided Fractionation

As a result of increasing interest in the plant kingdom as a potential source of new therapeutic agents, several techniques have been developed for the extraction and isolation of natural products. Recently, biological activity-guided fractionation and isolation linked with chromatographic separation techniques have been extensively applied. In this approach, the fractionation of extract is based on biological activity rather than a class of compound of interest which involves step-by-step separation of the plant extract. On the basis of physicochemical properties and screening for biological activity, further fractionation and screening are followed. In the first round, all the fractions are screened for biological activity, and only the fractions possessing significant activity are further processed until the achievement of the pure isolate, responsible for target biological activity. The chemical characterization and structural elucidation are performed after the identification of the active isolates [18,61,62]. The biological activity-guided isolation method has been used for the discovery of a variety of plant-derived natural products, including anticancer agents, camptothecin, and paclitaxel from *Camptotheca acuminata* and *Taxus brevifolia*, respectively [63]. Other natural products or modified forms of natural products include dopamine receptor agonist, apomorphine derived from morphine; tiotropium, used to treat chronic obstructive pulmonary disease derived from atropine; galantamine, a selective anticholinesterase obtained from *Galanthus nivalis* and arteether, an antimaerial agent derived from artemisinin [6].

The bioactivity-guided fractionation of natural products is a relatively new technique. In the experimental processes, mainly two approaches are used to isolate either known or unknown compounds which may be used as drugs or lead structures that may provide platforms in developing new analogues with better druggability. However, based on the circumstances other approaches can also be used. These two approaches are as follows.

### 4.1. Parallel Approach

**Figure 2:**
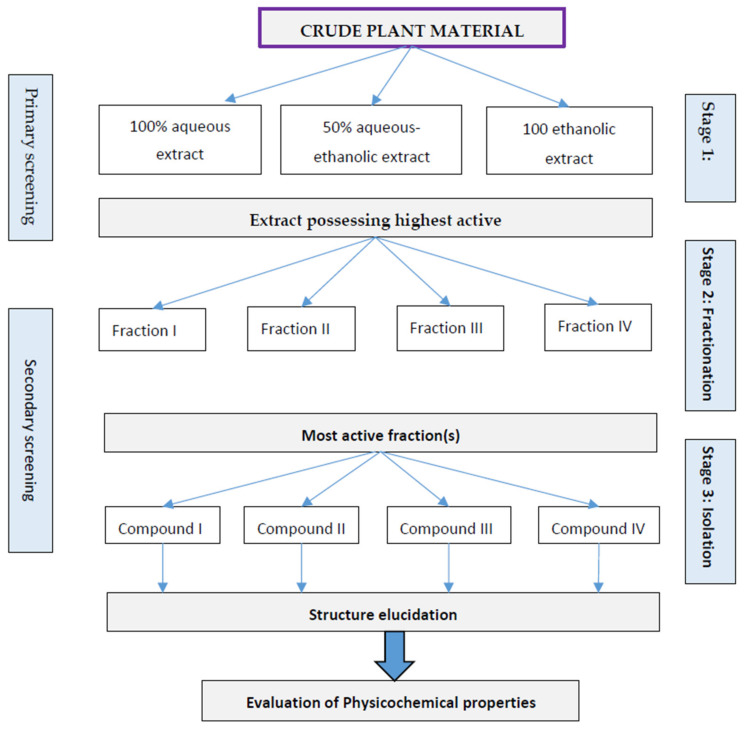
An outline of parallel approach for biological activity guided fractionation of plant extracts.

This approach is used when the selected plants are known for their biological activities from traditional or ethnopharmacological knowledge. The active compounds responsible for the target activity are isolated from the crude plant material as described in Figure 2. The extraction, isolation/purification is generally performed in the following three stages.

#### 4.1.1. Extraction

Initially, at least three fractions of extracts, for example, 100% aqueous, 100% ethanolic, and 50% aqueous −50% ethanol extracts are collected and evaluated for target biological activity in the primary screening.

#### 4.1.2. Fractionation

The most active extract(s) are extracted into sub-fractions in the sequence of decreasing polarity of a solvent such as butanol, chloroform, and hexane. The sub-fractions are further evaluated for biological activity.

#### 4.1.3. Isolation and Purification

The most active sub-fraction(s) obtained at stage 2 are subjected to chromatographic separation to isolate the compounds of interest. Each compound is purified through appropriate purification techniques such as column chromatography, preparative HPLC, etc., and screened for target biological activity. Chemical structures of the compounds exhibiting optimum biological activity are elucidated by using modern techniques such as NMR spectroscopy, Mass spectrometry, LC-MS, etc.

### 4.2. Sequential Approach

**Figure 3:**
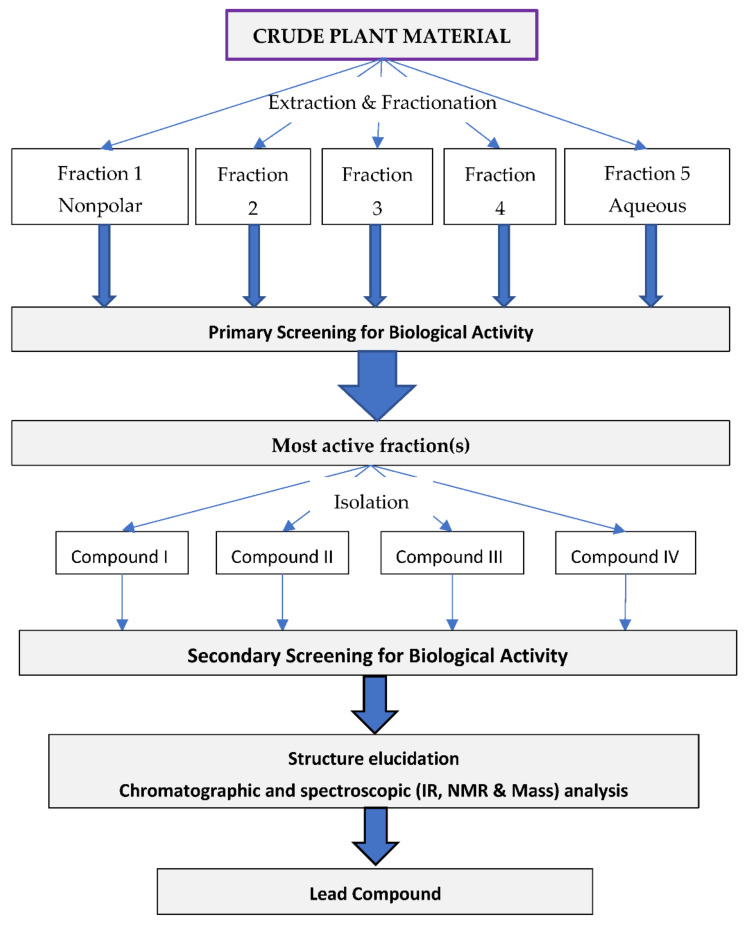
An outline of sequential approach for biological activity guided fractionation of plant extract.

This approach is mainly used for the plants selected by random selection strategy and the biological activity of the selected plant is not known. The extraction/fractionation, isolation, and biological screening processes used in this approach have been summarized in Figure 3. The experiment can be divided into two stages as follows.

#### 4.2.1. Extraction and Fractionation

In this stage, the extraction of the plant material and fractionation of the extracts take place simultaneously. The extractions are performed in the solvent systems of increasing polarity and fractions are collected in a sequential manner using petroleum ether, chloroform, ethyl acetate, ethanol, and water, for instance. All the fractions are screened for target biological activity.

#### 4.2.2. Isolation and Purification

The fractions from the stage 1 experiment possessing the highest biological activity are selected, and the compounds responsible for specific biological activity are isolated by following the techniques mentioned in the previous approach. The isolated compounds are analyzed for structure elucidation by using modern techniques such as LC-MS, NMR spectroscopy, FT-IR spectroscopy, and Mass spectrometry. As shown in Figure 3, the primary screening is done in the first stage to evaluate the efficacy, whereas the *secondary screening* is mainly oriented to the evaluation of the mechanism of action and may involve in-vitro screening at molecular levels.

In both approaches, the extraction of the plant material is performed by using a range of polar and non-polar solvents. However, their method of extraction and fractionation remained largely invariable. In general, the chemical classes of constituents present in the extract or fraction can be predicted depending on the polarity of the solvent used. Lipophilic compounds (low polarity constituents) such as oils, fatty acids steroids, hydrocarbons, and low polarity terpenoids are extracted in non-polar solvents such as n-hexane and ether; whereas, compounds with medium polarity such as phenolics and alkaloids are usually present in ethyl acetate and chloroform extracts. The highly oxygenated and high polarity compounds such as sugars, flavonoids, glycosidic alkaloids, and small carboxylic acids are generally obtained in aqueous or methanol/ethanol extracts.

### 4.3. Some Recent Experiment Using Bio-Activity-Guided Fractionation Technique

Tu et al. (2019) screened immunomodulatory isoflavone genistein against the production of pro-inflammatory cytokines (IL-6 and TNF-α) from the methanolic extract of *Uraria crinite* (L.) roots. They applied a bioactivity-guided fractionation approach in combination with NMR-based identification for isolation of the isoflavone and reported that genistein contributes to the immunomodulatory activity of the extract which may be helpful in standardization of the plant as functional food [64]. Nothias et al. (2018) applied a bioactive molecular networking approach in bioactivity-guided fractionation of antiviral compounds from previously investigated extract of *Euphorbia dendroides*, and isolated new constituents’ molecules that were not discovered by following the classical bioassay-guided fractionation approach. They used the mass spectrometric technique for dereplication of molecules prior to the isolation through molecular networking. They also implemented bioactivity score prediction to identify the biologically potential candidate molecules. The calculation of bioactivity score prediction was based on the relative abundance of the molecule in a fraction and the bioactivity level of the fractions [61].

In another study, a bioactivity-guided investigation was performed for fractionation of ethanolic extract of stem bark of *Pterocarpus dalbergioides* Roxb. and the butanol fraction exhibited potent anti-inflammatory and anti-hyperglycemic activities. Isoflavone genistein and phenolic acids including gallic acid and gentisic acid were isolated from the active fraction [65]. Iqbal et al. (2015) subjected the methanolic extract of *Ficus virens* bark to bioactivity-guided fractionation and a novel compound, n-octadecanyl-O-α-Dglucopyranosyl (6′→1″)-O-α-D-glucopyranoside was isolated. The compound was found to possess antihyperlipidemic activity through inhibition of HMG-Co reductase enzyme along with antioxidant potential. The new compound was further investigated by molecular docking analysis and ADME-T studies to evaluate pharmacokinetic profiles. The enzyme inhibitory potential was established through in-vivo antihyperlipidimic activity [66]. Recently, Abdallah et al. (2021) investigated the bioassay-guided fractionation substances of the stem bark extract of *Sclerocarya birrea*. The fractions were subjected to chronological partitioning and screened from antibacterial activity against *Salmonella typhi*. The ethyl acetate fraction of the extract was characterized and active principles were isolated using LC-MS/LC-HRMS technique. Vidarabine was found to be mainly responsible for the antimicrobial activity of the *S. birrea* stem bark [67]. In another recent study, Baldé et al. (2021) evaluated antiplasmodial and antimicrobial activities of *Terminalia albida* root. The samples were subjected to bioassay-guided isolation of phytocostituents using flash chromatography followed by characterization using NMR and HR-ESI-MS techniques. Among the 14 isolated compounds, pentolactone demonstrated the most significant activity against *Plasmodium falciparum*. Other compounds which exhibited antiplasmodial action include 3,4,3′-tri-O-methylellagic acid, triterpenes: arjunolic acid, arjungenin, and arjunic acid, and phenolic glycoside calophymembranside-B [68]. In addition to the above-mentioned reports, a number of investigations were performed by the natural chemists; where a bioactivity-guided fractionation approach was used for successful isolation of new or previously identified phytoconstituents [69,70,71,72,73,74].

## 5. Structure Elucidation of Isolated Compounds

Although a majority of pharmaceutical companies are showing insignificant interest in natural product research owing to their complex phytochemistry and difficulties in their access and supply [75], a substantial amount of work related to the discovery of therapeutic agents from natural sources are being carried out in academic set-ups and the technical complexities regarding isolation and structural elucidation of bioactive phytoconstituents are being overcome with the cooperation of the chemists globally. At present, spectroscopic analyses have become the main approach in the structural determination of phytochemicals. After a preliminary biological screening of extracts, the bioactive ones can be rapidly fractionated by high-performance liquid chromatography, subsequently, the chemical characterization of the fractions is performed by liquid chromatography-mass spectrometric (LC-MS) and nuclear magnetic resonance (NMR) spectroscopic analysis. After LC-MS analysis, the novel compounds in the isolates are first distinguished from the known compounds by comparing the MS data with those previously identified compounds available in the web libraries. Furthermore, adequate amounts of pure compounds can be readily isolated by HPLC through automated injection of extracts followed by the collection of fractions and their structural elucidation can be achieved through NMR and MS analysis. Consequently, the entire process starting from extraction to the isolation of pure natural compounds is now possible to achieve in days rather than months which was the case a few years ago [75,76,77]. Consequently, several pharmaceutical research institutions are presently working with this approach with the application of HTS technologies; their objective is to collect bioactive extracts, isolate the active phytoconstituents, and identify the potential lead molecules by exploring the effective pharmacophores from natural sources which may be further subjected to structural modification to develop analogues as good therapeutic agents.

LC-MS/MS is one of the most efficient techniques used for phytochemical profiling of the active fractions, which integrates good chromatographic separation efficiency with excellent characterizing capacity of mass spectrometry. Characterization of the structure of extracts, as well as pure bioactive molecules, are performed by the data obtained from a wide range of spectroscopic techniques such as FT-IR spectroscopy, NMR spectroscopy, and Mass spectrometry. In addition to these main techniques, data from X-ray diffraction, Optical rotatory dispersion, and chemical examination may also be helpful. A combination of HPLC with the above techniques has led to the development new technique useful in differentiating between known and unknown compounds and characterizing the full constituents of the natural products directly from the crude extract with a minimum amount of sample and time. The spectral data obtained from the instruments are interpreted to get information about the structure of the organic molecule under investigation.

**Figure 4:**
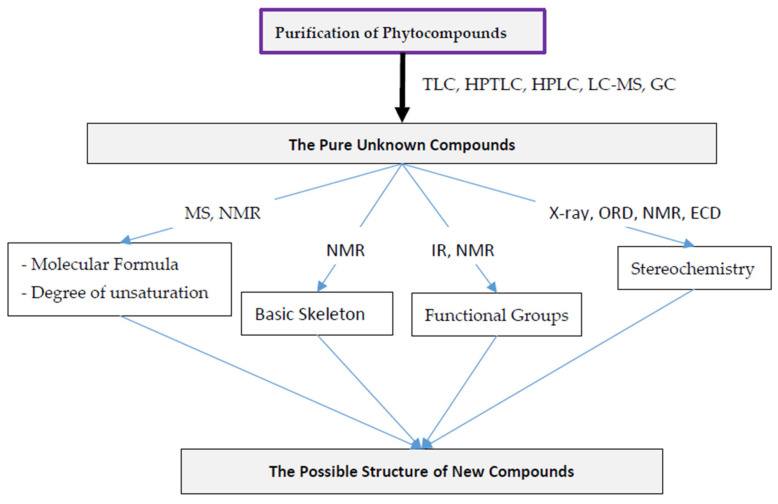
An outline for structural elucidation of new phytochemicals.

The development of efficient fractionation such as counter-current chromatography and emerging advancement in analytical techniques including NMR spectroscopy and mass spectrometry to isolate, purify and elucidate the structure of natural products is now allowing the natural product screening to fit in the timescale of high throughput screening [78,79,80]. It is now possible to isolate the bioactive compounds from the natural matrix and elucidate their structures within 4–5 weeks. Furthermore, complex structure-related issues can be solved with the use of significantly low quantities of a sample (sometimes less than 1 mg) [81]. To further enhance the productivity of natural product research several alternative techniques have also been explored. The chemical characterization using the analytical techniques is not only done for pure isolates, rather, crude extracts are also characterized for chemical composition mainly by using LC-MS/MS and GC-MS/MS techniques. Furthermore, preliminary NMR spectroscopic analysis can also be performed to explore the chemical constituents of the crude extract, even with complex composition. The NMR spectral data is helpful to identify the functional groups such as -OH, -CHO, -NH_2_, -NH-, OCH_3_, etc., and compounds including sugars, phenolics, steroids, terpenoids, and fatty acid esters. The presence of the most abundant chemical compounds or class of compounds in the extract can be identified, which helps to choose the most suitable separation method for further fractionation by either reverse or normal phase chromatography for instance [82]. The structural elucidation process of pure bioactive compounds from natural origins can be summarized as in Figure 4.

## 6. Biological Screening of Extracts/Fraction/Isolates

Generally, the natural products are screened for their biological activities on the basis of their reported ethnopharmacological and traditional uses. For instance, the medicinal plant traditionally used for the management of diabetes may be assayed for hypoglycemic effect and the traditional use is scientifically rationalized, once a very good ‘hit’ molecule has been achieved. However, most of the time the activity is not replicated in in-vitro screening. Most of the natural products are low-yielding, so the biological screening of such compounds can be performed by a series of bioassay methods that provide fast and sensitive results. These assays are performed by using several animal or human cell lines and microorganisms. A number of accurate and efficient instruments have been developed in this regard [83,84,85,86].

Although biological screening of extract and pure compounds from natural sources using animal models are still used, this approach suffers from demerits, including the requirement of large quantities of samples, lengthy and difficult experimental procedures, low sensitivity, and ethical consideration. Generally, the practical yield of bioactive pure compounds from natural sources is extremely low, and it is really difficult to supply them in adequate amounts needed for animal screening. On the other hand, potential hits may be regarded as unsafe on the basis of toxic effects observed in cell-based screening, which may have been revealing good safety profiles in the animal body due to detoxification in the liver [87].

The advancement of research in the field of life sciences has revealed a number of pathophysiological processes and mechanisms of drug actions, which led to the development of several cellular and molecular bioassay methods. A number of these bioassays fulfill the timescale requirement for HTS methods. The HTS methods could significantly minimize the amounts of test samples required for screening, needed in micrograms; permitting the assay of pure compounds isolated in very small quantities. Furthermore, the development of automation technologies, computer applications and software, and microfluid management has accelerated the identification of bioactive compounds (hits) as potential ‘lead’ molecules for target indication by making it possible to perform bioassay for thousands of samples within a short time period [88,89]. Incredible development in HTS technologies has been seen since the last 1–2 decades, and this has enormously increased the number of compounds to be screened from an average of 100,000 per year to approximately 100,000 per day. Consequently, the demand for structurally diversified molecules has greatly increased [90]. Furthermore, utilization of HTS methods has significantly enhanced the speed of screening through the acceleration of sample processing procedures including fast extraction and isolation of pure compounds from natural sources, and hence significantly extended the range of materials that can be assayed and the scope of drug bioassay. In contrast to synthetic compounds exhibiting biological activity in HTS, the molecules discovered from natural sources are more likely to possess optimum physicochemical properties of a drug and drug-like characteristics (druggability) [91].

After determining the biological profile of the new natural products; identification of their target of action in the biological system, physiological pathways they interact with and hence, mechanism of action should be established. These parameters require new approaches of modern drug discovery to be applied. The mechanism of action of natural products and their effects on the biological target can be determined by a battery of cell-based bioassays. The SAR studies provide preliminary information regarding the mode of drug-target interaction and identification of analogues showing higher potency than the parent compound. A compound exhibiting LC_50_ and IC_50_ values at micro or nano mole levels is considered to be potent. A more potent analogue needed to be administered in smaller amounts and hence, help to minimize the toxicities.

In a biological screening of drugs, the effects of solvent used on the dissociation of the drug and conformational or molecular arrangement should be taken into consideration. DMSO is one of the most commonly used solvents for this purpose, which is an aprotic solvent with intermediate polarity and capable to dissolve a variety of polar as well as non-polar compounds. However, due to the hygroscopic property of DMSO, the absorbed water sometimes reduces the solubility of non-polar compounds. Other organic solvents are usually not preferred due to their toxic effects on the test organisms or cells, in addition to poor miscibility with the assay media; however, the solvent controls are always used to cancel the toxic effects. Although, the in-vitro screening results are not always translated into in-vivo activity owing to a number of factors including dosage, solubility in a biological medium, membrane permeability, and biodegradation; a positive in-vivo activity of test compound is usually expected when it exhibited positive in-vitro results. However, the biological profile of a true drug candidate should not rely only on a single in-vivo screening, rather multiple screenings are necessary to confirm its efficacy [82]. The strengths and weaknesses of various biological screening models used for the natural product have been summarized in Table 3.

### Target-Based vs. Phenotype Screening Approaches

Since the last few decades, target-based screening has been considered the dominant approach in drug discovery. The starting point in this approach is a well-identified molecular target for drug interaction which has been considered to play important role in the disease. However, before the introduction of target-based screening, the chemical entities were evaluated through the phenotype screening approach, where a visible characteristic of an organism in a biological system, such as animals and cells, was investigated. The drug screening using a target-based approach is supported by advances in genomics, chemistry, and molecular biology; it provides the basis for measuring the efficacy and safety of drugs, dose setting, and selection of the patient population. The idea that rational and measurable progress would enhance the success rate and research productivity is among many factors that is responsible for the shifting of phenotype to target-based drug screening. Unfortunately, this change in approach could not greatly influence the success rate in the pharmaceutical industry. In the current scenario, owing to the scarcity of the new compounds acting on “essential” targets, there is a further revival of interest of researchers in the phenotypic approach, which relied on phenotypic measurement of responses and prior knowledge of specific drug targets or a hypothesis regarding their role in the disease is not essential. The screening approaches have been shifted to traditional phenotype and the lack of an established mechanism in this approach was considered as a deficiency, has now been observed as an opportunity. The development of genomic techniques has led to the identification of various new targets using optimized molecules as probes. Through the application of the phenotype approach, a number of new molecules have been identified as promising drugs, many without a known mode of action [92,93,94,95].

Both the approaches have their strengths and weaknesses and experience challenges. Although the drug target and mode of action is known, the challenge of target-based drug discovery is that it may not be able to capture the whole story. The observed drug efficacy may not be a result of the mechanism originally expected because a drug generally binds to more than one target to perform its action. Another challenge encountered is the in-vitro/in-vivo disconnect, where good efficacy on the target could not be translated to similar cellular activity in in-vivo screening. Although prior information on the mechanism of action is not required in phenotypic approaches, some understanding of biology is needed and at least the biomarker that translates to human disease should be identified. Moreover, it may be a risk to proceed a compound into development without some understanding of the mechanism to determine the dose-response relationship. The other major disadvantage of this approach is that the drug action may be the result of interaction at multiple targets leading to a poor structure-activity relationship in the optimization phase. Fortunately, various technologies such as affinity purification and biochemical fraction isolation are proved to be helpful. Additionally, this approach has also enabled the researchers to apply the latest molecular technologies of proteomics, network biology, and chemical biology [93,94].

Comparative studies revealed that the phenotypic approaches are more successful as far as small molecules, and first-in-class medicines are concerned. In a study, Swinney and Anthony (2011) [96] analyzed the comparative success of the two approaches and found that out of 259 agents approved by US FDA between 1999 and 2008, 75 were first–in–class drugs with new mechanisms, and of these, 28 first-in-class molecules came from phenotypic approach and 17 molecules were discovered through the target-based approach. The result indicated that the contribution of phenotypic screening to drug discovery is greater than the target-based approach, even though there was a major focus on the target-based screening during that period. Currently, natural products are being derived from both target-based and phenotype screening approaches. There are a large number of drugs derived from natural sources, where the target and mechanism of action were unknown at the time of discovery. Antimalarial drugs quinine and artemisinin are examples of such screening. The phenotypic screening approach has significantly accelerated the speed of the discovery of new therapeutic agents.

## 7. Molecular Modeling and Natural Product Database

The discovered bioactive natural products can be used as lead compounds for the optimization of structural features to develop new and more effective analogues by applying modern medicinal chemistry approaches, including molecular modeling and combinatorial chemistry. Furthermore, the natural products present along with other compounds as a family of structurally related molecules; therefore, a number of homologues may be obtained from one source which can provide SAR-related information. Since only a small proportion of the available plants have been studied for biological activities so far, the discovery of new leads from natural sources will continue, and they will be available for biological screening to find new therapeutic agents. In the modern drug discovery of natural products, the isolated new compounds possessing acceptable bioactivity are subjected to SAR studies and molecular modeling processes to design and develop analogues with more potency, fewer toxic effects, and better pharmacokinetic profiles. The study can also reveal that interaction with certain enzymes may influence the test compounds’ biological activity. The analogues with the best druggability may be synthesized in the laboratory and evaluated by a number of in-vitro and in-vivo biological assays [97]. The overall, process of design and development of analogues from a naturally isolated lead compound can be summarized as below.

### 7.1. In-Silico Ligand Construction and Preparation

Molecular modeling procedures require optimized 3D structures of ligands in PDB format. Natural product databases and other databases like PUBCHEM, ZINC are reliable sources for retrieving the structures of known natural compounds in different acceptable formats like SDF., mol., mol2, PDB, etc. [98]. Structures obtained must be optimized for geometry so as to possess minimum energy. Energy minimization can be performed prior to docking in docking software like AutoDock Vina and Discovery Studio, or independent structure building and optimization software like Chimera, Chem 3D Ultra, Avogadro, etc., can be applied [99,100].

### 7.2. Target Preparation

Preparing or downloading the 3D structures of target molecules such as proteins (example: human serum albumin), receptors (examples: PPAR-α and PPAR-γ), and enzymes (examples: cyclo-oxygenase, topoisomerase II, and protein kinase) from Protein Data Bank (PDB) and optimized for geometry and energy. The binding site to be defined and standard scores calculated for natural ligands present.

### 7.3. Docking

The 3D structures of natural products are docked against the target structure by using docking software and ranked according to the binding energy. Widely used docking tools include AutoDock, AutoDock Vina, FlexX, Discovery Studio, and MDock [100]. A pre-requisite for docking is the optimized 3D structures of the ligands and targets in PDB format. Docking utilizes several search algorithms, for example, the Lamarckian genetic algorithm for identifying the best binding conformation of the ligand. Post docking studies to analyze inter-molecular interactions are essential to substantiate the results obtained from docking.

### 7.4. Identification of Hit Molecule

After docking simulation, the results are analyzed and the top interactions are identified based on the energy scores. Usually, the top 10 scores are further subjected to molecular dynamics (MD) simulation studies. In this regard, two systems i.e., (I) Apo (uncomplexed) protein/receptor, (II) protein/receptor complexed with interacting compound (predicted by docking study) can be submitted to MD simulation. According to the ranking of ligands and their interactions with the target, the hit molecules having a high affinity towards the target are identified.

### 7.5. Optimization of Hits

The optimization process of hits is performed by observing better affinity towards the target by preparing various analogues of hits and the hits showing best affinity are developed and various drug-like properties viz; stability, pharmacokinetic and pharmacodynamic properties can be studied by using QSAR software [101,102].

**Figure 5:**
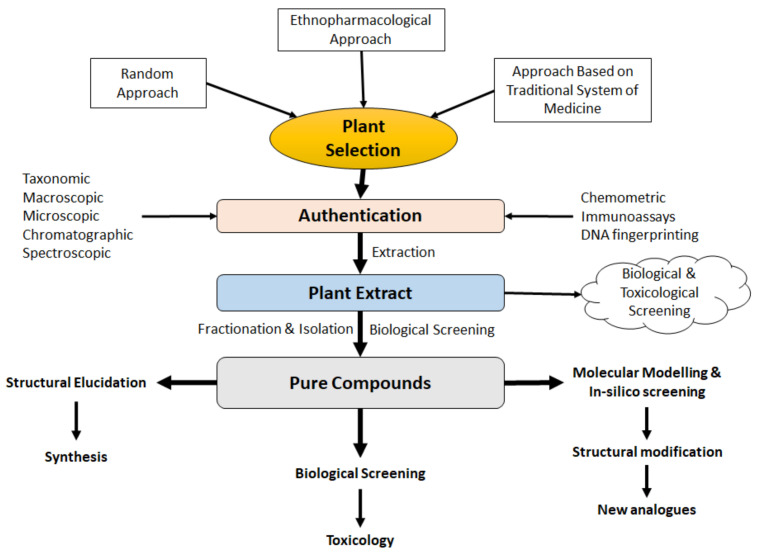
The overall approaches in modern drug discovery and development process from botanical sources.

Alternative to the extraction and biological assay of natural products, in modern drug discovery, a collection of a large number of compounds derived from natural sources possessing diverse chemical structures are tested through virtual screening or in-silico study. There are a number of such libraries held by academic institutions and research centers, holding up to millions of such structurally diverse compounds. Through the application of virtual screening, potential hits can be identified for a target biological activity and a good SAR can be established through a lead optimization process. In this way, virtual screening can filter down the number of compounds for the real test through bioassay [103,104]. However, the natural product database could provide only the structural information of the test compounds, the hit molecules should be physically available for the confirmation of the predicted new biological activity through relevant bioassay. Such compounds either can be synthesized in the laboratory or purchased if available from any commercial organization. DNP, Phytopure, ChemSpider, Natural Product Alert, Tim Tech Natural Products, etc., are some of the commonly available natural product libraries and databases [82]. The overall approach to drug discovery and development from natural sources has been summarized in Figure 5.

## 8. Bioactivity-Guided Fractionation Approach–Challenges and Advances

### 8.1. Identification of Bioactive Constituents

In natural product drug discovery, once the bioactive compound is identified, the plant extracts can be rationally designed with improved efficacy by modulating the concentrations of bioactive compounds; alternatively, the isolated compound may be used as a lead molecule for developing analogues with improved therapeutic potential. Bioactivity-guided fractionation is one of the most commonly used approaches in natural product drug discovery to identify the active molecules. As discussed above, in this approach extraction and biological screening proceed side-by-side. The fractions are collected, screened for biological activity and the process iteratively continued until the isolation and characterization of active constituents are performed [48,54,55,56,57,58,59]. However, loss of activity during the fractionation process is one of the major problems associated with this approach. Moreover, the fractionation is guided by bioactivity rather than structural information, the possibility of repeated isolation of already known compounds is very high [49,105]. Re-isolation of known compounds can be avoided through the application of the “dereplication” approach which involves the preliminary structural characterization for recognizing and discarding the known constituents. The dereplication is a relatively new approach that enables the effective use of compound discovery resources to prioritize the samples that are likely to contain new bioactive molecules [49]. In the dereplication process, the mass spectrometric, NMR, and UV-spectroscopic data of the constituents in a mixture are recorded and the compounds with known structures are searched by matching the spectral patterns in the dereplication database. Global Natural Social Molecular Networking (GNPS) is one such recently developed platform that enables spectral annotation and identification of related molecules using MS-MS molecular networking. The GNPS platform also provides the opportunity to researchers worldwide to share the raw MS-MS spectral data online and hence, allowing information sharing between the laboratories globally [49,106].

### 8.2. Identification of Synergists

Natural products generally consist of multiple constituents which can produce their action by interacting with a number of targets. Some of these constituents may act as additives or synergists to the therapeutic effects of the other bioactive constituents. Generally, synergistic constituents display no biological activity on their own; rather, they only increase the potency of bioactive molecules in combination [107]. Such compounds may be ignored if separated from active compounds during fractionation. Synergy-directed fractionation is a recent development in the bioactivity-guided fractionation approach, which allows the combination of chromatographic separation with synergy testing for the known active compound in the original extract. In this approach, extracts are tested for synergy, fractionated, the active fractions are again subjected to synergy testing and the process is repeated until the isolation of pure bioactive compounds. Through a combination of the fraction containing known active compounds and evaluation of combination effects, synergists could be identified. Three synergists for berberine have been identified by using this approach from *Hydrastis Canadensis*, which were overlooked in the conventional technique [108].

## 9. Conclusions

Historically, medicinal plants have been used for the treatment of various communicable and non-communicable diseases, and even today represent a rich source of important therapeutic agents as well as new lead compounds. A number of successful therapeutic agents have been obtained directly from plant sources or developed from naturally derived lead molecules. Revived scientific interest and research trends in plant-derived natural product drug discovery and development clearly indicated that it is one of the potential sources of new therapeutic agents in the future. In plant-based drug discovery and development research, plant metabolites are being optimized for developing potential analogues that can demonstrate desired safety and efficacy. Due to renewed interest of the medicinal chemists in natural product drug discovery, a number of new approaches accompanied by technological advancement for selection, identification, isolation, characterization, and biological screening of natural products have been developed. These new approaches could minimize the technical drawbacks associated with natural product development and address the challenges encountered in the discovery and development of new natural products owing to the complex behavior of natural products. The technological developments enabled the exploration of the profiles of complex phytoconstituents leading to the isolation or synthesis of a number of successful therapeutic drugs and novel lead compounds that can provide a core scaffold for future drugs. It is particularly important to adopt an interdisciplinary approach involving traditional and ethnopharmacological knowledge, phytochemistry, botany, analytical chemistry, suitable biological screening strategies, and modern drug development tools for successful results in this regard. In the future, the new strategies on natural product drug development will minimize the challenges and enhance the success rate; in the drug discovery process, the use of new molecules from plant origin and chemical libraries based on natural products will be increased. It may be a major contributor to new drug development and address global health challenges.

## Figures and Tables

**Table 1:** Important natural products derived from plant and microbial sources in the last few decades with their therapeutic indications and probable mechanism of actions.

Name of the Natural Compound	Botanical Source	Chemical Structure	Therapeutic Indication/Activities	Mechanism of Action	References
Drugs Derived from Plant Sources					
Arglabin	*Artemisia glabella*	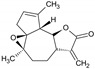	Anti-tumor	Inhibition of farnesyl transferase	[21,22]
Artemisinin	*Artemisia annua* L.	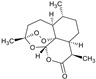	Treatment of malaria	Free radical formation that alkylate essential malarial proteins	[23]
Cannabidiol	*Cannabis sativa* L	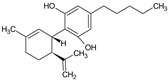	Anti-epileptic, anxiolytic, antipsychotic, and anticancer	Modulation of CB1, CB2, 5HT1A receptors in the CNS	[24]
Capsaicin	*Capsicum annum* L.; *C. minimum Mill.*	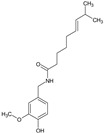	Chronic pain syndromes such as postherpetic neuralgia and musculoskeletal pain	Activates Transient receptor potential vanilloid 1 (TRPV1) in sensory nerves	[25]
Colchicine	*Colchicum* spp.	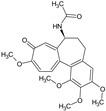	Gout	Prevents microtubule assembly and hence modulate multiple pro- and anti-inflammatory pathways	[26]
Curcumin	*Curcuma longa* L. (Turmeric)	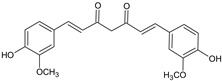	Antioxidant, anti-inflammatory, arthritis, metabolic syndrome and pain	Inhibition of NF-kB; scavenge reactive oxygen and nitrogen species; modulates the activities of GSH, catalase and SOD	[27]
Epigallocatechin-3-*O*-gallate (EGCG)	*Camellia sinensis* L. (Green tea)	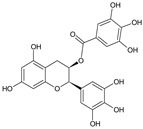	Anti-viral against a diverse family of DNA and RNA viruses; antibacterial and antifungal activities. Anticancer, anti-inflammatory and anti-diabetic activities	Alter or damage viral particle, primary target is viral membrane; disruption of lipid layer in bacterial cell wall; inhibits dihydrofolate reductase. Modulation of ROS production and inhibition of NF-kB signaling responsible for anticancer activity	[28,29]
Galantamine	*Galanthus caucasicus* Grossh.	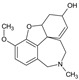	Dementia associated with Alzheimer’s disease	Reversible acetylcholinesterase inhibitor; modulation nicotinic acetylcholine receptor (nAChRs)	[30]
Genistein	*Genista tinctoria* L.	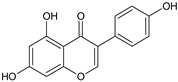	Anticancer, Alzheimer’s disease	Protein-tyrosine kinase inhibition, induction of apoptosis, cell cycle arrest, antimetastatic and antiangiogenic activity, antioxidant	[31]
Gossypol	*Gossypium hirsutum* L. (Cotton plant); *Thespesia populnea*	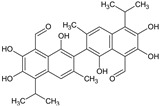	Anti-infertility/male contraceptive, Anticancer, antiviral, antimicrobial, antioxidant activities	Inhibit sperm production and motility; Bcl-2 inhibition; DNA polymerase and topoisomerase II inhibition; induce apoptosis	[32]
Ingenol mebutate	*Euphorbia peplus* L.	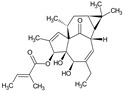	Actinic keratosis	Dual mechanism, Inducer of cell death necrosis and local pro-inflammatory response	[33]
β-Lapachone	*Tabebuia avellanedae* (Lapacho tree)	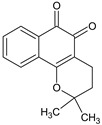	Variety of cancers, especially solid tumors, anti-*trypanosoma,* antimicrobial and antimalarial activities	Anticancer activity through formation of ROS in NQO1-positive cells, topoisomerase inhibition, mTOR pathway modulator	[34,35]
Masoprocol	*Larrea tridentate*	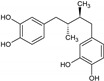	Antineoplastic agent used in cancer chemotherapy	5-Lipoxygenase inhibition	[36]
Omacetaxine mepesuccinate (Homoharringtonine)	*Cephalotaxus harringtonia*; *C. fortune*	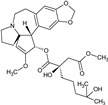	Anticancer agent; mainly chronic myeloid leukemia (CML)	Protein synthesis inhibition (prevent peptide elongation)	[37]
Paclitaxel	*Taxus brevifolia* Nutt.	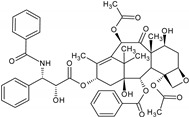	Cancer chemotherapy	Mitotic inhibitor	[36,38]
Podophyllotoxin	*Podophyllum emodi* Wall. and *P. peltatum* L.	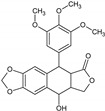	Antitumor	Polymerization of tubulin resulting in cell cycle arrest and suppress the formation of mitotic spindles microtubules	[39]
Quercetin	*Many sources including Allium cepa* L.; *Morus alba*; *Camellia sinensis*; *Moringa oleifera*; *Centella asiatica* etc.	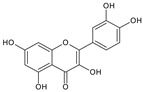	Antioxidant, anti-inflammatory, anticancer, cardiovascular protection; Alzheimer’s disease; anti-ulcer; antimicrobial; antiallergic	Inhibits cyclooxygenase and lipoxygenase; inhibits platelet aggregation; inhibit gastric secretion and lipid peroxidation; ROS generation and MicroRNA 21 elevation	[40]
Resveratrol	*Vitis vinifera* L	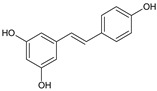	Chemopreventive and chemotherapeutic in different types of cancer. Also used as antidiabetic, in cardiovascular complications, metabolic syndromes, antioxidant.	Modulation of multiple molecular pathways involved in cancer and xenobiotic metabolism; reduce oxidative stress and inflammation; cell proliferation arrest; induce apoptosis	[34]
Drugs Derived from Microbial Sources					
Teixobactin	*Eleftheria terrace*	N-[N-Methyl-D-Phe-Ile-Ser-D-Gln-D-alle-Ile-Ser-]cyclo[D-Thr-Ala-[3-(2-iminoimid-azolidine-4 beta-yl)-Ala-]Ile-]	Antibacterial agent active against various gram-positive bacterial including vacomycin resistant enterococci and methicillin-resistant *S. aureus*	Inhibition of bacterial cell-wall sybthesis by binging to the synthesis building blocks lipid-II and lipid-III	[41]
Lodopyridone	*Saccharomonospora* sp.	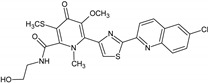	Anticancer	Cytotoxic to HCT-116 human colon cancer cells	[42]
Salinosporamide A	*Salinospora tropica*	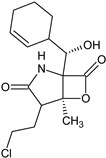	Anticancer	Inhibition of 20S Proteasome	[43]
Platensimycin	*Streptomyces platensis*	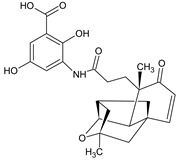	Antibiotic, active against various Gram-positive bacteria including resistant strains	Inhibition of fatty acid synthesis in cell membrane through inhibition of β-ketoacy synthases I/II (FabF/B)	[44]
Platencin	*Streptomyces platensis*	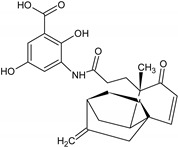	Antibiotic, active against various Gram-positive bacteria including resistant strains	Inhibition of fatty acid synthesis in cell membrane through inhibition of β-ketoacy synthases I/II (FabF/B)	[44]
Cryptophycin	Cyanobacteria *Nostoc* sp.	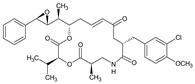	Anticancer	Inhibiotion of cell division by depletion of microtubule through interaction with tubulin	[45]
Daptomycin	*Streptomyces roseosporus*	-	Systemic and life-threatening infection caused Gram-positive bacteria	Disruption of bacterial cell-membrane function	[46]
Retapamulin	*Pleurotus mutilins*	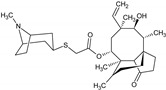	Antibacterial used to treat topical skin infection impetigo	Inhibition of bacterial protein synthesis by binding to 50s ribosome	[47]

**Table 2:** Ethnopharmacological/traditional approach versus random screening approach of plant selection.

	Ethnopharmacological/Traditional Approach	Random Selection Approach
Characteristics	- Traditional or ethnopharmacological knowledge/application is the basis for plant candidate selection and pharmacological assay. It involves the observation, description and experimental analysis of traditionally used plant materials. - The *traditional system of medicine*, such as TCM and Ayurveda, possess well established written knowledge about medicinal plants and regularly revised. - The ethnopharmacological knowledge is easily accessible.	- Plant candidates for natural product discovery are randomly selected, mainly based on their availability.
Strengths	- Comparatively higher success rate. - Based on scientific disciplines including chemistry, botany, pharmacology, biochemistry, history, anthropology et.	- Extremely advantageous, when plant species from a region of high biodiversity has to be screened. - The selected samples has the potential of identification of unexpected biological activities and novel structures. - Can be applied for both general and focused pharmacological screening.
Weaknesses/Challenges	- Permits are needed for the collection and investigation of plant candidate; even may provoke legal-issues with the ethical groups or the country in which the traditional knowledge was originated, - Traditional systems such as Ayurveda and TCM use multicomponent mixtures as formulation and the identification of active constituents out of these mixtures are complicated due to complexity and synergistic effects - The concept of health and disease in traditional medicine widely deviate the modern concepts. For example TCM is highly influenced by Chinese philosophy. This may complicate the correct interpretation of the ethnopharmacological information. - Holistic and personalized approaches of these systems are difficult to access by current bioassay methods.	- Lower rate of success in comparison to ethnophramcological approach. - Flawed in the sense that there is no idea of bioactivity. - The pharmacololgical screening used for randomly selected samples are of small or medium throughput and the test samples (extracts, fractions or pure constituents) availability is low limiting the number of bioassays that can be done.
Examples	- Galegine isolated from *Galega officinallis* L. inspired the synthesis of metformin and other biguanidines antidiabetics; papaverine from *Papaver somniferum* L.; quinine from Peruvian *Cinchona* bark inspired the synthesis of chloroquine and mefloquine [20,48]; artemisinine from TCM herb *A. annua* led to the development of artemether [49]; andrographolide from *Andrographis paniculata*; Berbarine from *Berberis aristata* etc [18].	35,000 plant species screened through random selection between 1960 to 1980 leading to discovery of paclitaxel and camptothecin [18].

**Table 3:** Strengths and weaknesses of various biological screening models used for natural products.

Screening Models	Strengths	Weaknesses
In-vivo animal models	Physiological similarities to humans; pathophysiological relevance is high; activity on the level of whole organism and transgenic models may be generated.	Require to manage animal facility; need larger amounts of test samples; ethical consideration; low-throughput; may be species related differences.
In-vitro cellular target-based assays	Known molecular target; no need to determine the mechanism of action separately; efficacy of hits at cellular level, high-throughput	Observed efficacy may not be a result of the mechanism originally expected because a drug generally bind at more than one target; may not be able to reflect whole mechanism of the hits; no assurance for in-vivo efficacy; requirement of cell culture facility
In-vitro phenotype cell-based assays	Potential to discover new molecular target; medium to high-throughput; efficacy of hits at cellular level	No assurance for in-vivo efficacy; requirement of cell culture facility; identification of molecular target may need great effort; possibility of poor structure activity relationship of hits in the optimization phase
In-vitro assays with isolated proteins	No animal or cell culture facilities required; high-throughput screening	Hits may be unable to reach the target for interaction into cells or in-vivo (hits with low bioavailability)
In-situ/ex-vivo isolated tissues or organs	Higher-throughput than animal models; good pathophysiological relevance	Lower-throughput than cell-based bioassays; ethical consideration; short life of isolated tissues and organs

## Data Availability

Not applicable.
